# Segmental Abdominal Paresis Attributed to Herpes Zoster Infection Mimicking an Abdominal Hernia: An Interesting Case From a Surgical Unit of a Tertiary Healthcare Center

**DOI:** 10.7759/cureus.51728

**Published:** 2024-01-05

**Authors:** Aditya Sharma, Satyendra K Singh, Vivek Srivastava, Arvind Pratap, Mumtaz A Ansari

**Affiliations:** 1 Department of General Surgery, Institute of Medical Sciences, Banaras Hindu University, Varanasi, IND; 2 Department of Dermatology & Venereology, Institute of Medical Sciences, Banaras Hindu University, Varanasi, IND

**Keywords:** postherpetic pseudohernia, neurological complication, acyclovir therapy, abdominal hernia, herpes zoster virus

## Abstract

The varicella-zoster virus reactivates to cause herpes zoster, commonly referred to as shingles. Shingles traditionally manifest as itchy vesicles in a dermatomal distribution, accompanied by related constitutional symptoms in immunocompetent patients. Usually, the rash resolves completely in seven to ten days. Herpetic neuralgia is the most typical herpes zoster consequence. Around 1% to 5% of individuals have motor impairments, with Ramsay-Hunt syndrome being the most prevalent ailment.

Additional problems encompass abdominal pseudohernia, paralytic ileus/colonic pseudo-obstruction, hemidiaphragm paralysis, bladder dysfunction, localized paresis, constipation, and visceral neuropathy. Herpes zoster infection typically involves the posterior root ganglia, and most of the symptoms are sensory. Motor involvement can occur in the same distribution but is relatively uncommon. Segmental zoster paresis is a rare motor complication of herpes zoster, mimicking an abdominal hernia, which has an incidence of approximately 0.7%, but it needs no surgery different from the real abdominal wall hernia.

In this case report, we describe a patient who, three weeks after developing a herpes zoster rash, acquired an abdominal protrusion, i.e., herpes-induced pseudohernia.

## Introduction

The varicella-zoster virus (VZV), which is activated in the dorsal-root ganglia, causes the neurological condition known as herpes zoster [1]. While motor neuropathy is a rare side effect of the herpes zoster virus infection, sensory abnormalities are the most common manifestation of the disease [2]. One of the rare motor consequences of segmental zoster is abdominal paresis, which has an incidence of approximately 0.7% [3]. The same resembles an abdominal hernia; however, unlike a true abdominal wall hernia, it does not require surgery. 

Since the posterior root ganglia are frequently involved in herpes zoster infections, the majority of symptoms are sensory. Although it is not common, motor involvement may occur in the same distribution. Segmental zoster paresis primarily affects the face and limbs, and most cases have been reported in middle-aged and older people [1]. There are only a few case reports that describe abdominal wall weakening manifesting as flank or abdominal bulges that mimic hernias [1-4].

The majority of these reports showed that the bulge and weakness resolve on their own after proper medical therapy. It is crucial to recognize this entity because it emphasizes that surgery is not necessary and that it may be reversible. We present a 38-year-old female patient who had a typical herpes zoster rash along with an abdominal protrusion.

## Case presentation

**Figure 1:**
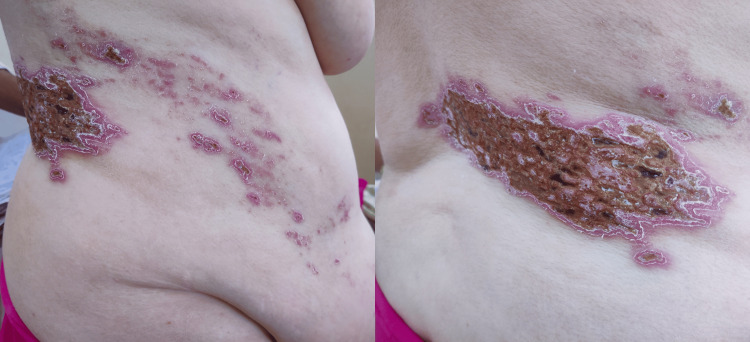
A clinical picture showing a healed herpetic skin rash with dimensions of 20 × 8 cm, extending from the back to the bilateral flanks and umbilical region.

A 38-year-old female presented at our healthcare facility with chief complaints of a widespread rash that had spread throughout the affected region (abdomen, flanks, and back) and had been present for two weeks following being bitten by an infant. The abdominal wall protrusion occurred concurrently, and it was not associated with any pain. She had no prior medical history of any comorbidities or any surgical intervention. A healed herpetic skin rash with dimensions of 20 × 8 cm was noticed extending from the back to the bilateral flanks and umbilical region, as shown in Figure 1.

**Figure 2:**
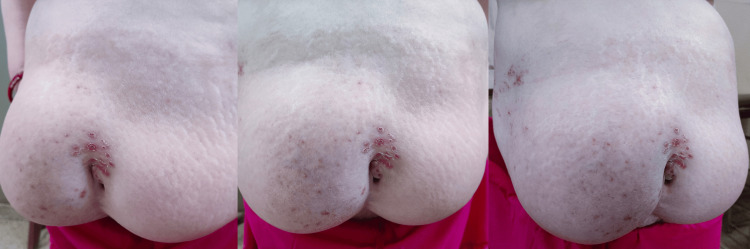
A clinical picture showing bilateral abdominal wall bulging that became more prominent and noticeable with increased abdominal pressure.

Muscle strength and limb activity were both within normal limits. There was bilateral abdominal bulging that became more prominent and noticeable with increased abdominal pressure, as shown in Figure 2.

After seeking a dermatologist's opinion, it was found that the patient had segmental herpes zoster with abdominal paralysis, a motor complication of the disease that mimics an abdominal hernia, rather than the abdominal wall defect or mass that was initially suspected. Oral acyclovir (800 mg five times a day for seven days), pregabalin (75 mg once daily for 14 days), and methylcobalamin (1500 mcg immediate release once daily for 14 days) were recommended. 

Within days after treatment, she reported improvement in the associated pain but continued to have a persistent bulge. She was discharged to follow up with her treating dermatologist, who noted continued improvement in pain and the associated abdominal protrusion. The patient recovered spontaneously four months after the onset of the disease.

## Discussion

Broadbent (1886) described the first example of motor impairment following herpes zoster [4]. There is currently no standard terminology for it, and in many medical publications, it is referred to as a zoster lumbar hernia or zoster pseudo hernia [5]. The more precise description for this entity would be segmental zoster abdominal paresis [4,5].

We found only 73 cases that had previously been reported by examining the PubMed database as of December 2023. The patients ranged in age from 45 to 84, with an average age of 67.8 years and a male-to-female ratio of 7:1. At an average of 2.9 weeks, segmental zoster abdominal paresis developed 1-6 weeks following the rash's onset. The most commonly impacted dermatome is T11, which is followed by T12 and T10. One of the primary neurological consequences of herpes zoster is altered sensory perception; however, motor neurons can also be affected as well, leading to paralysis of the diaphragm, muscles of the face, limbs, or abdomen, or in certain circumstances, visceral involvement [6].

Although the precise underlying mechanism is unknown, it is believed to involve viral infection at the spinal cord's anterior horn level as a result of the VZV spreading from the dorsal root ganglia to the brain. Pathologically, ganglion lesions, along with significant neuritis and degeneration of the motor and sensory roots, may account for the disease's electrophysiological findings. 

Unless it is contraindicated (infants and immunocompromised individuals), all seronegative subjects who come into contact with a newborn infected should be administered immunization (two doses separated by at least six weeks) within 72 hours of the infectious encounter, especially adolescents and adults who are significantly at risk of a severe disease presentation [3,7]. 

The primary approach to diagnosis is clinical, depending on the moment of the onset of abdominal distension and the presence of Herpes zoster. A physical examination may show that segmental reflexes are absent or minimal [2,3]. A confirmation of the diagnosis can be achieved with an electroneuromyographic test; however, only 35 percent of cases show changes. Abdominal computed tomography rules out the presence of an abdominal growth or hernia and reveals a thinner abdominal wall. Nuclear magnetic resonance imaging using gadolinium-diethylenetriamine penta-acetic acid (DTPA) can be used to exclude spinal nerve root compression and help determine the extent of inflammation [5].

The diagnosis of herpes zoster-related abdominal pseudohernia relies on multiple factors. Firstly, just because there isn't a rash does not imply that the physician should rule it out. In about 10% of cases, the bulging occurs before the herpetic rash, even though the illness is commonly referred to as postherpetic abdominal pseudohernia [7]. Second, a focused history is crucial, for example, to rule out tick exposure, child bites (as seen in the present case), and neurologic problems linked to Borrelia. Third, if close follow-up does not reveal delayed onset, imaging studies, particularly magnetic resonance imaging, may be considered to screen for mechanical compression of thoracic nerve roots [8].

The diagnostic combination is an abdominal wall bulge accompanied by a herpetic rash. Close observation may be beneficial since the rash could appear after the swelling. The differential diagnosis should include conditions including lumbar hernia, polyradiculoneuropathy, diabetic neuropathy, and syringomyelia that manifest as changes in the innervation of the muscles of the abdominal wall and can result in pseudohernia [2,8].

The course of treatment is the same as with herpes zoster: antiviral medications and, if necessary, analgesics. Although there is little evidence to support the use of these treatments, short courses of corticosteroids and other vitamin formulations have also been used for their anti-inflammatory effects and to help regenerate injured nerve fibers [3,7,8].

For motor weakness, the prognosis is generally favorable; between two and eighteen months, 55% to 75% of cases will recover completely or almost completely [1,3,7]. Constipation is the most frequent side effect of pseudohernia; however, in 19.4% of cases, paralytic ileus and voiding difficulties have also been reported [6,8].

## Conclusions

In conclusion, abdominal pseudohernia is an uncommon side effect of herpes simplex that often has a favorable prognosis. Despite the clinical nature of the presumed diagnosis, a noninvasive imaging test should be carried out to rule out a true hernia. Both an abdominal bulge and an abdominal wall herniation are caused by postherpetic pseudohernia. This illness, which essentially involves abdominal muscular paresis, is one of the neurological aftereffects of herpes zoster. Since herpes zoster is a widespread illness, there seem to be a lot of individuals with pseudohernia, even though this condition is not well known. Due to its lack of awareness, postherpetic pseudohernia might be confused with abdominal wall herniation, and if a proper workup is not done, the patient might land up in hernia repair surgeries, which he or she might not require. Surgeons and dermatologists, in particular, need to be aware of this phenomenon in order to treat patients effectively. 
